# GeneDig: a web application for accessing genomic and bioinformatics knowledge

**DOI:** 10.1186/s12859-015-0497-0

**Published:** 2015-02-28

**Authors:** Radu M Suciu, Emir Aydin, Brian E Chen

**Affiliations:** Centre for Research in Neuroscience, Research Institute of the McGill University Health Centre, Montreal General Hospital, 1650 Cedar Ave, L7-224, Montréal, H3G 1A4, Québec Canada; Departments of Medicine and Neurology & Neurosurgery, McGill University, Montréal, Québec Canada

**Keywords:** Data integration, Genomics, Bioinformatics, Molecular biology, Genome editing, Database, Software

## Abstract

**Background:**

With the exponential increase and widespread availability of genomic, transcriptomic, and proteomic data, accessing these ‘-omics’ data is becoming increasingly difficult. The current resources for accessing and analyzing these data have been created to perform highly specific functions intended for specialists, and thus typically emphasize functionality over user experience.

**Results:**

We have developed a web-based application, GeneDig.org, that allows any general user access to genomic information with ease and efficiency. GeneDig allows for searching and browsing genes and genomes, while a dynamic navigator displays genomic, RNA, and protein information simultaneously for co-navigation. We demonstrate that our application allows more than five times faster and efficient access to genomic information than any currently available methods.

**Conclusion:**

We have developed GeneDig as a platform for bioinformatics integration focused on usability as its central design. This platform will introduce genomic navigation to broader audiences while aiding the bioinformatics analyses performed in everyday biology research.

**Electronic supplementary material:**

The online version of this article (doi:10.1186/s12859-015-0497-0) contains supplementary material, which is available to authorized users.

## Background

The primary impediments to accessing genomics and bioinformatics data are the volume of information available and the level of background knowledge required to navigate data repositories [1-4]. The end result is that much of the bioinformatics data remains inaccessible to average end-users such as biologists or physicians, or end-users spend inordinate amounts of time navigating these resources [5-7]. Therefore, we sought to create an intuitive web-based application that will allow any general user more efficient access to genetic information, and in doing so, translate the massive and complex amounts of genomics and bioinformatics data into a simple and easy-to-navigate web resource.

## Implementation

We have developed GeneDig as a platform for entry into publicly available genomic data with broad expansion possibilities. The genomes of any sequenced organism can be searched or browsed. Our genomics navigator takes advantage of recent developments in web technologies such as HTML5 to provide the most robust and smooth user experience possible. Previous genome browsers have avoided these technologies for the sake of compatibility, but as the adoption rate of modern web browsers increases, we believe that by leveraging these new functionalities we will deliver a better user experience to generate a larger user base that would normally be discouraged in accessing genomics. Our focus at this stage of GeneDig as a platform is on broad appeal and robust operation rather than overwhelming users with a large range of highly specialized technical functions. We use Backbone.js in a model-view-controller framework for JavaScript to separate the program logic from the graphical user interface. In addition to helping organize our code, this makes it easier to implement new features and have other bioinformaticians add tools to our platform. Backbone also provides built-in state management to allow users to save the exact state of the genome navigator and webpage at any time by copying the uniform resource locator (URL, e.g., web address) so that anyone with the necessary permissions may access it at a later date.

To create an intuitive genome browsing experience, we were challenged with allowing the user to seamlessly navigate across the full genome scale from the chromosome map to specific nucleotide sequences. This difference in scales is several hundred thousand-fold. To accomplish this, we used the canvas element standardized in the HTML5 specification. Canvas provides high performance methods to manipulate and display data without any observable lag between user actions. This allows for an intuitive method of changing the field of view by grabbing the data to pan and zoom, providing continuous viewing of data without reloading the webpage or jarring the user with a loss of reference point or orientation. To our knowledge, no other genome browser can seamlessly visualize data from the chromosome map to nucleotide zoom levels [5,6,8-10] (Table 1). This approach to genome interaction is thus more efficient, and the possibilities for expansion are far greater when integrated with HTML5 text editing features and visual overlays.

**Table 1:** **Comparison of genomic portals and web-based genome browsers**

	**No page reloads for genome viewing?**	**Seamless zoom from chromosome to nucleotide views?**	**All sequenced organisms browsable?**	**RNA and Protein sequences on one page?**	**Protein domain information?**		**Copy and paste of sequences?**	**Multiple languages?**	**Search enabled?**	**Average time to locate results**
**GeneDig**	✔	✔	✔	✔	✔		✔	✔	✔	30 sec
Ensembl [6]	∅	∅	✔	P	✔		∅	∅	✔	2 min
NCBI [5]	✔	∅	✔	P	∅		∅	∅	✔	1 min
UCSC Genome Browser [8]	∅	∅	∅	P	∅		∅	∅	✔	3 min
GBrowse [10]	✔	∅	∅	∅	∅		∅	∅	∅	∅
JBrowse [9]	✔	∅	∅	∅	∅		∅	∅	∅	∅

Feature assessments were performed at the time of writing. P: Protein track available. Users were timed performing 5 specific genomic tasks (Additional file 1: Figure S1).

### Molecular biology focus

Biology researchers use bioinformatics data almost daily as part of experimental procedure, but the current resources that exist require a high level of expertise to operate. For example, in the realm of analysis tools or algorithms, these resources are targeted towards high-level users and specialize only in the features within their designers’ expertise. Because they are scattered across the web, this also makes it difficult for general users to stay abreast of advances in bioinformatics analysis. Thus, when end-users are able to uncover these helpful resources, they are confronted with field-specific syntax when inputting sequences, in addition to unfamiliar features, settings, and terminology. If researchers need to take data from one analysis website to another (e.g., genomic DNA sequences to a protein domain predictor website), they must navigate different interfaces between the websites and again different syntaxes, field codes, and other idiosyncrasies between the three or more websites that biologists often have open at a time. However, because no alternative exists for many of these resources, biologists are willing to spend any amount of time required to master these tools to fulfill a research need.

A common biology paradigm is structure-function analysis of a molecule of interest involving the manipulation of DNA sequences encoding the amino acid sequences of specific protein domains. To do this the researcher must: identify the specific protein domain of interest, then the amino acid sequence that corresponds to this protein region (Structure), and then DNA sequence to be mutated that corresponds to these amino acids, then several rounds of molecular cloning including PCR, restriction digests, and bacterial transformations, until the modified DNA is re-inserted into the model system for analysis (Function). This example is commonplace in biology, biochemistry, chemistry, and biomedical research labs across the world, but currently requires dedicated software suites and bioinformatics analysis websites all used in conjunction to aid in the different steps of the procedure. Molecular biology manipulations have pervaded almost every experiment in biology research, and all of these experiments benefit from easier access to DNA, RNA, and protein sequence data and analysis tools.

Our interface is designed for the general user, but several features will also specifically aid molecular biology experiments. These include displaying the RNA and protein sequence data of the gene in focus in the genome browser, as well as displaying the protein domain information, all in one page. Advances in genome editing tools and rapid adoption of these techniques [11] necessitates a clear visualization and understanding of the precise relationship between genomic DNA to protein domain. Thus, co-navigation and co-highlighting of DNA, RNA, and amino acid sequences were implemented to aid in these structure-function and genome editing experiments. Importantly, all sequence data can be highlighted for copy/paste, including genomic sequences within the genome browser. Of the current genome browsers that can zoom in to the nucleotide sequence, these are rendered as images and are thus not able to be selected [5,8,9] (Table 1). Additionally, rather than being able to browse only specific examples of organisms or single chromosomes, all sequenced organisms can be searched and browsed on GeneDig, without the need to download and install the application locally.

## Results and discussion

**Figure 1:**
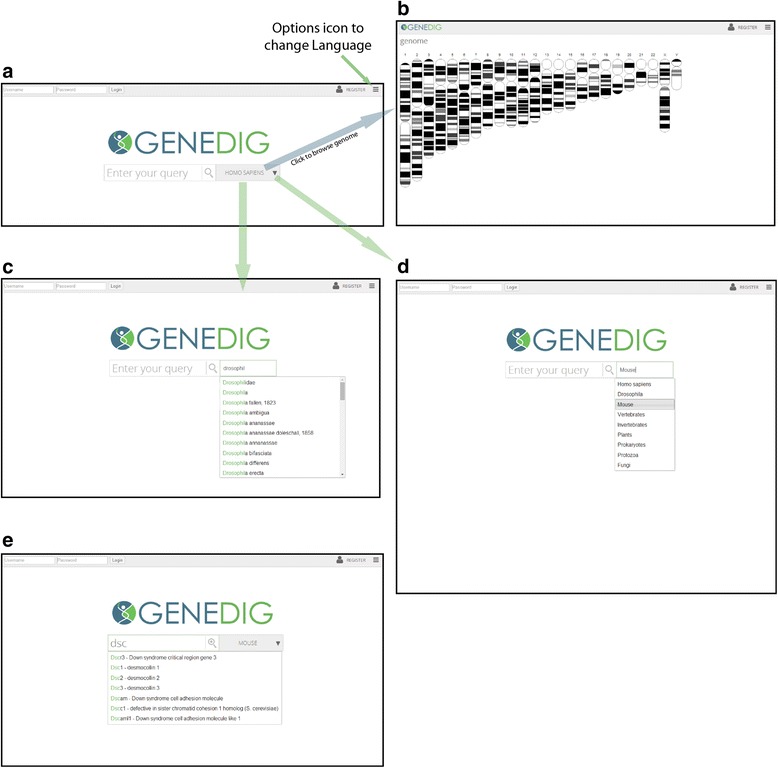
**GeneDig provides easy access into genomics.** Searching for genes and organisms within the web app is aided by search suggestions and drop down menus. **a**, The landing page contains a single search box with optional additional features for advanced users. The human genome is searched for by default. GeneDig is multi-lingual accessible, and the language can be changed by moving to the upper right icon and clicking on Language to display a list of more than 15 different languages. **b**, Genomes can be directly browsed by simply clicking on the organism name, which takes the user to the chromosome map. **c**, Searches can be filtered by typing in an animal name or group name (e.g., mammals). Search suggestions are built into the filter for difficult taxonomic names, and common names of animals and misspellings can be entered. **d**, Specific collections of genomes can be selected in a drop down menu (e.g., Vertebrates). **e**, Gene searches are simplified by allowing for disease names, gene names, and other genomic features such as gene ID number.

GeneDig is freely available at http://genedig.org. On the main page, a single search box is shown with the organism *Homo sapiens* as the default search species (Figure 1). GeneDig is multilingual accessible and available in 17 different languages. The language can be changed by moving the cursor over the Options icon in the upper right hand corner (Figure 1a), and clicking “Language”. Searches typed into the search box can include gene names, gene IDs, diseases, or traits (Figure 1). Genomes can also be browsed directly by clicking on the organism name, which will take the user to the organism’s chromosome map (Figure 1b). Clicking on a chromosome will take the user to the GeneDig navigator with the chromosome gene structure in view.

**Figure 2:**
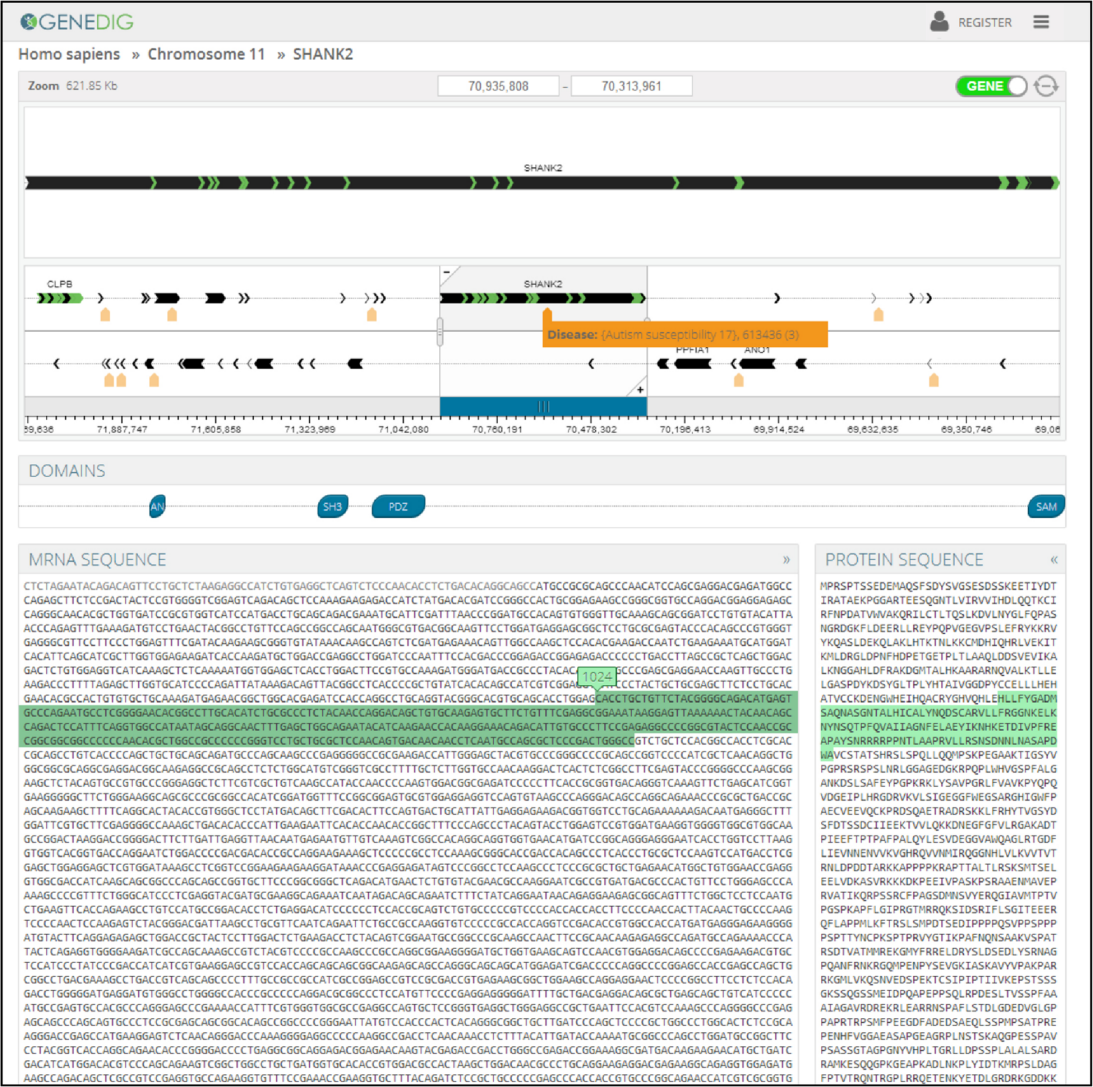
**GeneDig allows for seamless browsing of genomes, RNA, and protein sequences.** The GeneDig navigator consists of the genome browser (top window) that displays gene organization, protein domain structure information (Domains window), mRNA sequence data, and amino acid sequence data. Black arrows within the genome browser represent individual genes, with the green arrow lines representing the exons within each gene. Diseases associated with any genes in view are indicated by an orange tab. Corresponding DNA, RNA, amino acid sequences, and protein domains can be co-highlighted for copy and paste (e.g., hovering over nucleotides in the RNA sequence highlights the corresponding amino acid sequence). RNA untranslated regions are in grey and the coding sequence is in black.

All sequenced genomes are available for searching. The organism’s genome to be searched can be changed by clicking on the down arrow next to the organism name, and then either choosing from a drop-down list of commonly searched organisms or collections of organisms (e.g., Vertebrates), or by typing in the common name or Latin name of an organism (Figures 1c, d). Search results return a list of relevant genes with summary information for each gene, such as the gene name, location, gene size, RNA size, protein size, and disease associations. Clicking on a gene search result takes the user to the GeneDig navigator (Figure 2), with the selected gene in view.

The GeneDig navigator allows for genome and DNA browsing (window at the top of the webpage), RNA, and amino acid sequence co-navigation, and protein domain viewing (Figure 2). In the genome browser, different genes within the genome can be browsed from either the positive or negative DNA strand. Genes are shown in black arrows, and exons within a gene are labeled in green. Diseases and traits associated with genes are indicated with orange and blue tags, respectively (Figure 2). Using this visual overlay structure, our platform can be expanded to overlay other “-omics” data, such as annotations and tracks [12]. The genome can be browsed by sliding the panner window around, or by clicking and grabbing the main window to scroll left and right. Clicking on a gene in view will snap to the gene to display the RNA, amino acid, and protein domain information. To display the entire DNA nucleotide sequence for a gene in view, click on the toggle icon in the upper right hand corner of the genome browser window to switch between gene schematic and gene sequence views. The field of view can be changed directly by entering in coordinates into the nucleotide coordinate display boxes, or by moving the cursor over the Zoom information and selecting from 1 kilobase (kb), 10 kb, 100 kb, 1 Mb, 100 Mb, and All (whole chromosome) scale views. Zooming in or out can also be performed by using the mouse wheel, or by grabbing the side of the panner window to narrow the field of view that the genome browser main window is displaying (Figure 2). The genome browser allows for seamless zooming from displaying all the genes on a chromosome to single DNA nucleotide sequences.

To visualize the relationship between DNA sequences and its protein product, the GeneDig navigator co-highlights DNA, RNA, and amino acid sequences that are selected by the cursor (Figure 2). For example, clicking and selecting any DNA exon (i.e., coding) sequence, or RNA sequence, or amino acid sequence will highlight the corresponding other sequences. Hovering the cursor over the mRNA sequence will co-highlight the specific corresponding amino acid sequence and vice versa. Hovering or clicking on specific protein domains in the protein domain schematic will co-highlight the corresponding RNA and amino acid sequences. This is demonstrated by moving the cursor from N-terminus of the protein schematic to the C-terminus as the highlighted RNA and amino acid sequences move from top to bottom. These features allow the direct visualization of how RNA codon sequences relate to amino acids, and which RNA sequences are translated and untranslated (untranslated RNA regions are displayed in grey). Currently, the RNA and protein sequence isoforms that are displayed are the first isoforms (e.g., isoform A) listed in the NCBI database, where all of our sequence data are derived from [5]. In the future, we will implement an interactive visual navigation to select alternate isoforms that can be produced from a gene, and integrate other public “-omics” databases.

GeneDig’s novel features aid molecular biology experiments in particular by allowing direct visualization of genomic DNA and RNA (i.e., cDNA in experiments) sequences frequently targeted or modified. For example, for genome editing experiments, it is often important to know where the genomic DNA sequence corresponding to a specific protein domain is, and whether it spans multiple exons and how large the intervening introns are. In another example in genome editing, foreign sequences are often inserted before or after the untranslated RNA regions to tag proteins. Similarly in cDNA manipulations, insertions and edits of specific sequences that correspond to protein coding and non-coding untranslated sequences are required. No other genomic application allows for direct visualization of this central dogma of molecular biology, the intertwined relationship between DNA, RNA, and protein.

**Figure 3:**
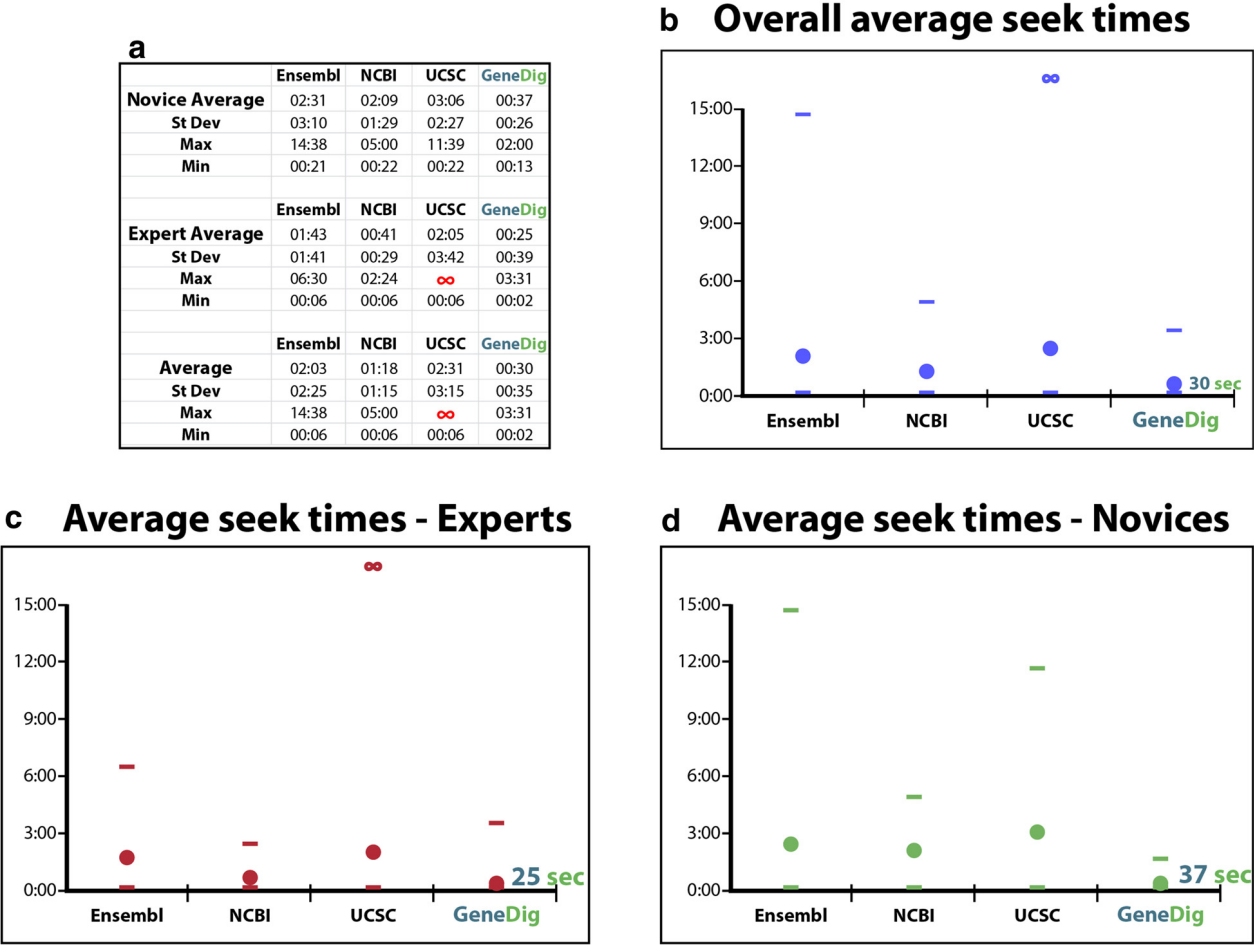
**GeneDig allows for much faster access to bioinformatics data. a**, Average participants’ seek times for the GeneDig Challenge using different genomics portals. Experts (n = 6) were classified as medical and graduate students, post-docs, physicians, and professors who regularly used one or more genomics portals. Novices (n = 7) consisted of high school and undergraduate students, or any other users who were not familiar with any genomics web resources. Times were capped by participants at 6, 7, or 20 minutes (designated as ∞), for any task that participants gave up on. **b - d**, Graphs of average times overall, Expert times, and Novice times. Average times are shown as filled circles, horizontal lines represent maximum and minimum times. Novices were as fast or faster than Experts at performing genomic search tasks.

Our interface is designed so that any general user that requires information about specific genes or diseases will be able to rapidly find it (Figure 3). Similar to any application that provides easier access to large volumes of complex data, our technology can be easily adopted for educational purposes. Through universal design and offering direct navigation and visualization of the core relationships in biology between DNA, RNA, and protein, GeneDig can be used as an educational tool for students to learn about genetics, genomics, and bioinformatics. Thus, we have also made our website available in more than 15 languages including the top 10 languages of internet users.

GeneDig will aid genomic inquiries in a variety of ways, such as in identifying whether a mutation in a DNA sequence is within a protein coding region, or which RNA sequences a protein domain corresponds to, for experimental manipulations. To provide quantitative evidence of the increase in efficiency provided by using GeneDig, we designed 5 tasks to represent the most frequent and basic information most users would seek when performing basic biology experiments (Additional file 1: Figure S1). Because we could not predict *a priori* how long each participant would take for each task at each portal, each task was designed to be a simplistic version of real queries to not overly burden participants, and to be easily understandable. However, these 5 tasks in aggregate accurately represent the daily bioinformatics queries that occur in biology and biomedical research labs, by genetic counselors and physicians, and individually are near identical to classroom exercises performed by university students. Participants were asked to time their ability to find the data requested by each task, using three genomic portals (NCBI, Ensembl, and the UCSC portal) compared to GeneDig. Participants were polled from any background (science, medicine, and non-science) and expertise level, from high school student to principal investigator. We found that seek times for GeneDig were five times faster than the three major genomics portals (Figure 3a). The fastest individual seek time for the NCBI was comparable to GeneDig’s, but overall was still significantly slower than GeneDig (p < 0.0001) (Figure 3b). Importantly, when comparing expert versus novice seek times, we found that by using GeneDig, novices were as fast or faster than experts using other genomics portals (Figures 3c, d). In other words, our results show that GeneDig allows people with no previous genomics experience whatsoever to be as efficient as experts at finding bioinformatics data.

## Conclusions

Our web application lowers the barriers to entry into accessing and using genomic information. Making genomics more accessible will significantly enhance biology research, science education, and health care. As personalized medicine initiatives begin to provide patients with information on their own genomes, we expect that our tool will also help educate the general public to perform their own investigations into questions of health and disease on the genomic and proteomic levels. With the advances in DNA sequencing and DNA synthesis, genome editing tools, and the potential of personalized genomics, the implications for a user-friendly genomics portal and navigator that the general public will be able to adopt are significant.

## Availability and requirements

**Project name:** GeneDig

**Project home page:**http://genedig.org

**Operating system:** Platform independent

**Programming language:** HTML, JavaScript

**Other requirements:** A web browser that supports HTML5, such as Google Chrome 10.0 and higher, Internet Explorer 10 and higher, Mozilla Firefox 10.0 and higher, Opera 12 and higher, and Safari 6.0 and higher.

**License:** None

**Any restrictions to use by non-academics:** Freely available

## Additional file

Additional file 1: Figure S1. The GeneDig challenge quantifies efficient access to genomics and bioinformatics data. The 5 challenges were designed to replicate the standard tasks most experimental biology and biomedical labs perform when requiring the use of bioinformatics data, without overly burdening the participants’ time.
