# Supplementary material for: GeneDig: a web application for accessing genomic and bioinformatics knowledge
Source: BMC Bioinformatics. 2015 Feb 28;16(1):67. doi: 10.1186/s12859-015-0497-0 (PMC4349619; doi:10.1186/s12859-015-0497-0)
Supplement: Additional file 1: Figure S1. — The GeneDig challenge quantifies efficient access to genomics and bioinformatics data. The 5 challenges were designed to replicate the standard tasks most experimental biology and biomedical labs perform when requiring the use of bioinformatics data, without overly burdening the participants’ time. [file 12859_2015_497_MOESM1_ESM.docx]

**Supplementary Information**

**GeneDig: A Web Application for Accessing Genomic and Bioinformatics Knowledge**

Radu M. Suciu^1^, Emir Aydin^1^, Brian E. Chen^1,2,*^

**^1^** Centre for Research in Neuroscience, Research Institute of the McGill University Health Centre, Montréal, Québec, Canada.

**^2^** Departments of Medicine and Neurology & Neurosurgery, McGill University, Montréal, Québec, Canada.

*Correspondence to: [brian.chen@mcgill.ca](mailto:brian.chen@mcgill.ca)

**
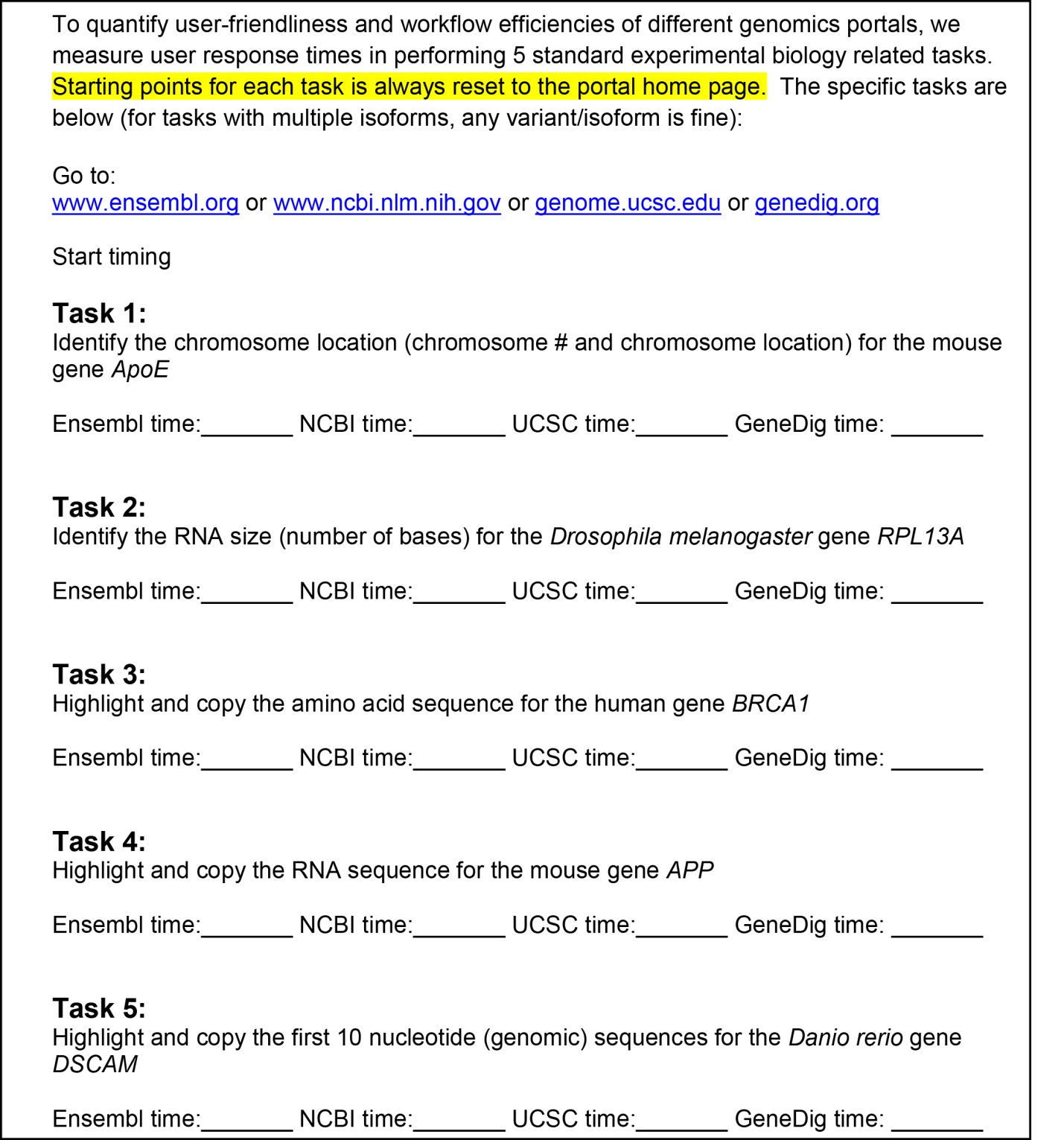
**

**Supplementary Figure 1 |** **The GeneDig challenge was created to quantify efficient access to genomics and bioinformatics data.** The 5 challenges were designed to replicate the standard tasks most experimental biology and biomedical labs perform when requiring the use of bioinformatics data, without overly burdening the participants’ time.
